# Microbial metabolites in the pathogenesis of periodontal diseases: a narrative review

**DOI:** 10.3389/froh.2023.1210200

**Published:** 2023-06-14

**Authors:** Amina Basic, Gunnar Dahlén

**Affiliations:** Department of Oral Microbiology and Immunology, Institution of Odontology, the Sahlgrenska Academy, University of Gothenburg, Gothenburg, Sweden

**Keywords:** host-microbe interplay, periodontal diseases, inflammation, bacterial metabolites, proteolytic activity, hydrogen sulfide, ammonia, short chain fatty acids

## Abstract

The purpose of this narrative review is to highlight the importance of microbial metabolites in the pathogenesis of periodontal diseases. These diseases, involving gingivitis and periodontitis are inflammatory conditions initiated and maintained by the polymicrobial dental plaque/biofilm. Gingivitis is a reversible inflammatory condition while periodontitis involves also irreversible destruction of the periodontal tissues including the alveolar bone. The inflammatory response of the host is a natural reaction to the formation of plaque and the continuous release of metabolic waste products. The microorganisms grow in a nutritious and shielded niche in the periodontal pocket, protected from natural cleaning forces such as saliva. It is a paradox that the consequences of the enhanced inflammatory reaction also enable more slow-growing, fastidious, anaerobic bacteria, with often complex metabolic pathways, to colonize and thrive. Based on complex food chains, nutrient networks and bacterial interactions, a diverse microbial community is formed and established in the gingival pocket. This microbiota is dominated by anaerobic, often motile, Gram-negatives with proteolytic metabolism. Although this alternation in bacterial composition often is considered pathologic, it is a natural development that is promoted by ecological factors and not necessarily a true “dysbiosis”. Normal commensals are adapting to the gingival crevice when tooth cleaning procedures are absent. The proteolytic metabolism is highly complex and involves a number of metabolic pathways with production of a cascade of metabolites in an unspecific manner. The metabolites involve short chain fatty acids (SCFAs; formic, acetic, propionic, butyric, and valeric acid), amines (indole, scatole, cadaverine, putrescine, spermine, spermidine) and gases (NH_3_, CO, NO, H_2_S, H_2_). A homeostatic condition is often present between the colonizers and the host response, where continuous metabolic fluctuations are balanced by the inflammatory response. While it is well established that the effect of the dental biofilm on the host response and tissue repair is mediated by microbial metabolites, the mechanisms behind the tissue destruction (loss of clinical attachment and bone) are still poorly understood. Studies addressing the functions of the microbiota, the metabolites, and how they interplay with host tissues and cells, are therefore warranted.

## 1. Introduction

The microbiota that colonizes various parts of the human body, such as skin, genitals and gastrointestinal tract, lives in a symbiotic relationship with the host (1). Each part of the gastrointestinal tract, e.g., oral cavity, pharynx, esophagus, stomach, small and large intestine, has its own site-specific core microbiome, which is regulated by the physiological and other environmental factors that characterize every ecological unit (habitat). Collectively, these microbial habitats and their genomes constitute the human microbiome, with characteristic compositions for each niche in which the microorganisms live in a symbiotic and homeostatic condition with the host, and together constitute the symbiont. The main benefits of this host-microbiome symbiosis are, among others, resistance to colonization by exogenous pathogens, support in host defense functions and anti-inflammatory properties, antioxidant activity, support in the regulation of the cardiovascular system, additional metabolic potential, and support to maintain a healthy digestive tract (2). The tissue response to the colonizers along the entire gastrointestinal tract is a normal and valuable reaction to the microbial load in each habitat. The degree of mucosal lymphoid tissue manifestation beneath the epithelial barrier of the mucosal membranes is balanced against the metabolic activity and growth of the microorganisms in each habitat (3).

The ecology of the dental biofilm (dental plaque) is developed by an establishment of a highly diversified microbiota, ecologically termed as a climax community (4). This ecology, in the gingival crevice, develops around all teeth, in all humans and even animals (with a similar anatomy and physiology) and the microbiota is typical for this niche. It is true that in populations/individuals that practice dental cleaning the bacterial load and diversity is lower, however, the number of individuals with a gingiva fulfilling the criteria for being “healthy”, with a bleeding on probing < 10%, is a minority (5). Classical experimental gingivitis studies also confirm that the establishment of a climax community within the gingival crevice in adults takes less than 14 days, although there is no time limit for the continuous and life-long maturation process, with further adaptation and reorganization of a dynamic subgingival plaque community (6–9). The response of the host, seen clinically as an inflammatory response (gingivitis), should be regarded as a natural protecting response (a normal condition) rather than pathologic, and is balanced (homeostasis) against the microbial activity and release of metabolic products from the dental biofilm in the gingival crevice.

Numerous studies have been carried out in the past in search for specific species/genera or combinations/communities that are associated with destructive periodontal disease (10–12). Unfortunately, most studies are cross-sectional using advanced molecular methods with a limited number of subjects. Further, the specific details of the sampling strategy and the clinical status of the patients can be elusive to obtain. Several species (putative periodontal pathogens, key stone periodontal pathogens) have been associated with deep (>6 mm) periodontal pockets, however, their roles in the disease process are still far from established. Morphological studies report on bacterial masses in the periodontal pockets, with an adherent part against the root surface and a non-adherent part, including a high number of motile bacteria, densely packed in the most apical part of the pocket (13, 14). Studies on the composition of the periodontal pocket microbiome/microbiota generally show a predominance of Gram-negative strictly anaerobic species using protein fermentation as their main nutritional pathway in a slightly alkaline environment (2, 15). The periodontitis microbiota is associated with an overgrowth of some microorganisms such as *Porphyromonas* spp. and *Treponema* spp. (spirochetes), resulting in an increased microbial diversity in periodontitis compared to periodontal health (16). It is possible that these bacteria constitute a higher relative risk for further periodontal breakdown, but they are commonly present in high numbers also in shallow pockets and in gingivitis cases as well, and their impact on the periodontal disease progression is therefore not well established. The term “dysbiosis” is sometimes used to refer to the changes in the composition of the microbiota in particular niches (for example, those found in deep periodontal pockets). It should, however, be borne in mind that such proportional changes, although often considered pathologic, are more likely an adaptation of oral commensals to a modified microenvironment. Dysbiosis is mainly a term that normally refers to microbial imbalance or maladaptation within the microbiome (16, 17).

In recent years the function e.g., metabolic activities of the dental biofilm community rather than its composition have gained an increasing interest in both caries and periodontitis research (18–22). The dental biofilm metabolism is extremely complex and contrasting findings from *in vitro* and cell culture studies are common. The metabolism of the subgingival dental biofilm and the interaction of bacterial metabolites with the host tissues is still poorly understood (23).

This paper reviews the metabolic processes in the subgingival dental biofilm and its complex interaction with the gingival tissues and inflammatory cells of the host, supporting the view of an alternative perspective on destructive periodontal disease.

## 2. The metabolism of oral biofilms

**Figure 1:**
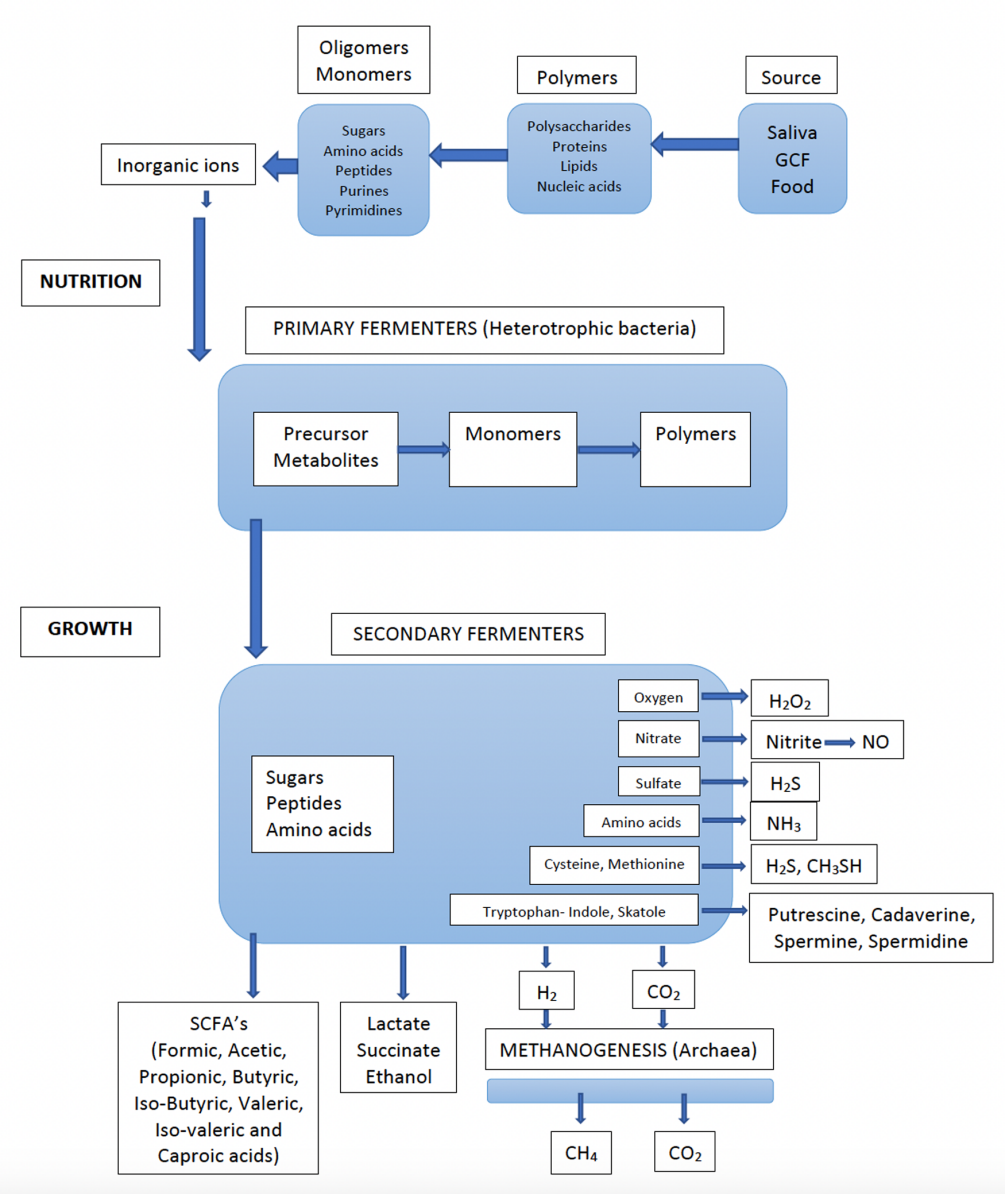
Flow of nutrients through an anaerobic microbial community (e.g., dental biofilm), whereby all the nutrients are converted into methane (CH_4_) and carbon dioxide (CO_2_) by the concerted actions of primary and secondary fermenters, sulfate reducers and methanogenic archaea. Detailed information on bacterial species involved in various metabolic processes are found in Table 1. (Adapted from Carlsson J. Growth and nutritions as ecological factors; In Kuramitsu H.K. Ellen R.P (Eds), Oral bacterial ecology. The molecular basis. Horizon Scientific Press, Norfolk, UK. Chapter 2, pages 68-130*).*

The oral cavity and its microbiota should be seen as the portal entry and the functional starting point of the gastrointestinal tract. A major beneficial activity of the gastrointestinal microbiota is the fermentation of food. The processes of degradation of the polymeric components (polysaccharides, proteins, lipids, and nucleic acids) into smaller molecules (oligomers and monomers), together with inorganic ions, provide the nutrients for host cells (Figure 1). They also provide the nutrients and cell materials (polymers) for the bacteria that colonize the entire gastrointestinal tract. This is a major function also for the oral microbiota. Although there are substantial differences between the gastrointestinal microbiota and the microbiota on the teeth and tongue, there are also some similarities, e.g., complex compositions, strong microbial interactions, and the degradation of nutrients (24). Firmicutes (mainly streptococci), which are biochemically the most active species in the oral cavity due to their potent ability to ferment both carbohydrates and peptides, represents the predominant phylum in the oral resident microbiota.

**Table 1:** Bacterial metabolites produced during the proteolytic, amino acid degradative, and other degradative processes that occur in the periodontal pockets.

Metabolite	Produced by proteolysis of	Bacterial species involved	Bacterial enzymes involved	Levels detected in/from the subgingival pockets	Potential impacts on bacteria and biofilm	Potential impact on human host
Hydrogen peroxide (H_2_O_2_)	O_2_ Pyruvate	*Streptococcus* spp.	Oxidases	*	Regulatory function (59)	Inhibition of inflammasomes (60)
**Acids**						
SCFA: Formic acid Acetic acid Propionic acid Butyric acid Isobutyric acid Valeric acid Isovaleric acid	Carbohydrates Proteins/amino acids (18, 61)	*Actinomyces* spp. *Bacteroides* spp. *Corynebacteria* spp. *Eubacterium* spp. *Fusobacterium* spp. (62–65) *Haemophilus* spp. Megasphaera spp. *Neisseria* spp. *Propionibacterium* *Prevotella spp.* (62, 65) *Porphyromonas* spp. (62, 65, 66) *Rothia* spp.	Transcarboxylase	3.85 mM (67) 9.5 mM (68) 11.68 mM (67) 3.11 mM (67) 3.11 mM (67)	Antibacterial activity (69, 70)	Pro-inflammatory (71) Anti-inflammatory (70) Neutrophil-specific chemoattractant (63, 66) Impact on brain function (72)
Other carboxylic acids: Succinic acid Caproic acid Isocaproic acid Phenylacetic acid	Carbohydrates Proteins/amino acids	*Porphyromonas gingivalis* (65) *Prevotella intermedia* (65)	Phosphoenolpyruvate carboxylase/carboxykinase (18)	1.24 mM (67)	Antibacterial activity (73)	Neutrophil-specific chemoattractant (66)
**Bioactive amines**						
Indole Skatole Cadaverine Putrescine Spermine Spermidine	Tryptophan Lysine Ornithine Arginine	*Fusobacterium* spp. (64, 74–77) *Lactobacillus* spp. (75) *Prevotella* spp. (75) *Porphyromonas* spp. (75, 78) *Streptococcus* spp. (75) *Treponema denticola* (78)	Tryptophanase (76)	Up to 1.74 mM (79) 2.76 mM 0.64 mM 0.58 mM (80)	Increased resistance to antibiotics (81) Effects on biofilm formation, cell metabolism, cell differentiation, plasmid stability, drug resistance, and signaling (78, 82–84)	Bacterial virulence (78) Toxic (85) Cell physiology (84, 86)
**Gases**						
Ammonia (NH_3_)	Proteins/amino acids Arginine (61) Lysine Methionine Cysteine Cystine Tryptophan Urea	*Fusobacterium* spp. (65) *Porphyromonas* spp. (65) *Prevotella* spp. (65) *Tannerella* spp. *Treponema* spp. *Lactobacillus* spp. *Peptostreptococcus* spp. *Helicobacter pylori* *Campylobacter ureolyticus* *Haemophilus parainfluenzae* *Streptococcus* spp. *Actinomyces* spp. *Staphylococcus* spp. *Rothia dentocariosa*	Arginine dihydrolase system Tryptophanase (76) Urease	* Urea levels up to 53 mM (87)	Increased resistance to antibiotics (88) Impaired function of neutrophils (89)	Toxic (90) Impaired function of neutrophils (91)
Carbon monoxide (CO)	Heme (92)	*Streptococcus mitis* (93)	Heme oxygenases	*	Antimicrobial (94)	Gasotransmitter (95, 96) Toxic (97)
Carbon dioxide (CO_2_)	Carbohydrates Proteins/amino acids Arginine Methionine Cysteine Urea	*Streptococcus* spp. *Lactobacillus* spp.	Arginine dihydrolase system Urease	*	Stimulus for the growth of most anaerobes (98)	Toxic (99)
Hydrogen gas (H_2_)	Carbohydrates Proteins/amino-acids	*Campylobacter* spp.	Hydrogenases	*	Bacterial survival and growth	Anti-inflammatory (100)
Hydrogen sulfide (H_2_S)	Cysteine Sulfate	*Fusobacterium* spp. (101, 102) *Parvimonas micra* *Porphyromonas* spp. *Prevotella intermedia* *Treponema denticola* *Streptococcus anginosus* *Desulfobacter* spp. (103, 104) *Desulfovibrio* spp. *Desulfomicrobium orale*	Cysteine desulfhydrases Cysteine lyases Cysteine synthases (105–107)	Up to 0.1 mM (108) Up to 1.5 mM (109) Up to 1.9 mM (110)	Harmful in high concentrations (111) Increased resistance to antibiotics (112, 113) Increased resistance to immune-mediated killing (114, 115) Protection from oxidative stress (116)	Toxic at high concentrations (117–119) Pro-inflammatory (120, 121) Anti-inflammatory (122) Gasotransmitter (95, 96)
Methane (CH_4_)	Hydrogen gas (H_2_) Carbon dioxide (CO_2_) Acetate Methylamine (123)	*Campylobacter* spp. Archaea *Methanobrevibacter*	Methyl coenzyme M reductase	*	*	Associated with severe colonic diseases (124)
Methyl mercaptan (CH_3_SH)	Methionine	*Fusobacterium* spp. (101)	L-methionine-γ-lyase (76)	Up to 0.16 mM (110)	Altered biofilm composition (125)	Decreased synthesis of collagen (126) Pro-inflammatory (125, 127)
Nitric oxide (NO)	Nitrate (NO_3_^−^)	*Veillonella* spp. *Rothia* spp. *Actinomyces* spp.	Nitrate reductase	Up to 0.13 mM for teeth and 0.17 mM for implants (128)	Bactericidal (129) Increased resistance to antibiotics (130) Antibacterial activity (131) Increased biofilm dispersal (117)	Gasotransmitter (95, 96)

* No data found.

The fermentation process in the oral microbiota follows two main pathways, one for carbohydrate (sugars and sugar alcohols) and one for proteins (peptides and amino acids), both described in detail by Takahashi (18). Nutrients from ingested food, especially mono- and di-saccharides, are readily available and efficiently fermented by streptococci, also under anaerobic conditions (19). The oral microbiota is momentarily activated through the carbohydrate-rich diet, especially through intake of sugars (mono and disaccharides). This can easily be followed by measurement of acid formation (mainly lactic acid) and the subsequent pH drop (Stephan curve) (25). For the resting microbiota on mucosal membranes and dental plaque (e.g., at night during sleep), under starvation conditions, the nutrients comprise salivary glycoproteins that are degraded into oligomeric molecules through the fermentation of both carbohydrates and proteins. It is important to emphasize that even during resting and starving conditions the microbiota of various oral niches are still metabolizing and growing and continuously fluctuating in activity (26, 27).

A major metabolic activity that takes place is the oxidation processes that results in the production of hydrogen peroxide (H_2_O_2_). This process is mainly occurring in the presence of oxygen in the dental biofilm (Figure 1) (18). The main producers of H_2_O_2_ are *Streptococcus* spp. and it is suggested that that their production of H_2_O_2_ has an important regulatory function against anaerobes to become established on exposed surfaces such as the mucosal membranes and teeth surfaces. Thus, H_2_O_2_ is regulating the dental plaque since certain bacteria lack protection from peroxides and oxygen radicals. There are, however, many both facultative and anaerobic bacteria, that are protected from oxygen-free radicals by enzymatic antioxidants such as superoxide dismutase, glutathione peroxidase and catalase (28). Since peroxides also have toxic effects on host epithelial and other cells, saliva contains lacto-peroxidase, which convert thiocyanate (SCN^−^) to hypothiocyanite (OSCN^−^), which is strongly antibacterial. The source of cyanate is through the diet, but cyanate is present in significant concentrations also in blood. It is therefore likely that this conversion of SCN^−^ to OSCN^−^ also takes place in the gingival pocket by myeloperoxidase from neutrophils. Increasing inflammation may thus efficiently degrade the peroxides and thus work as antioxidants and keep the anaerobiosis in the periodontal pocket. Interestingly, smokers seem to have higher concentration of cyanate in serum and may therefore contribute to more anaerobic conditions, favoring anaerobic bacteria (29). However, little is known about the importance of myeloperoxidase in anaerobic conditions of the periodontal pocket and whether the conversion of SCN^−^ to OSCN^−^ have any role in the periodontal pocket ecology or if this reaction is mainly occurring in supragingival areas.

Interestingly, oral rinsing with urea, momentarily leads to degradation of the urea to ammonia (NH_3_) and a pH raise followed by a neutralization back to initial status within 30 min (30, 31). The degradation of urea is due to urease which is strongly produced by some Gram-negative bacteria such as *Campylobacter ureolyticus*, *Helicobacter pylori* and *Haemophilus parainfluenzae* and moderately by some Gram-positive bacteria such as *Streptococcus* spp. and *Actinomyces* spp (32).

Nitrite (NO^−^), is another metabolite formed in the oral cavity through the reduction of nitrate (NO_3_^−^) by the bacterial enzyme nitrate reductase (33). Numerous oral bacterial species such as *Veillonella* spp., *Rothia* spp., *Neisseria* spp, and *Corynebacterium* spp. share this ability to reduce nitrate. In acidic environment (stomach acid) nitrite is further reduced to nitric oxide (NO), which is strongly linked to the cardiovascular system and regulation of blood pressure (34–38). Thus, a diet rich in vegetables (that have high nitrate content), in combination with nitrate reducing oral bacteria have indirectly linked the oral nitrate-nitrite-nitric oxide pathway to systemic NO dependent effects and cardiovascular health in previous studies (39–45). Whether the reduction of nitrite to NO takes place also locally in the acidic conditions (at sugar intake) at the surface of teeth is unknown but likely (46, 47). This potential trait of the dental biofilm has been claimed to counteract the caries process (48–50). Significant amounts of nitrite, suggested to have a local antimicrobial effect, is formed after intake of nitrate rich foods. Intragastric NO production has been measured in humans in expelled air but will also contain NO produced locally in the oral cavity (51, 52). It is likely that the formation of nitrite and NO mainly occurs at the tongue, but its potential antibacterial effect here is controversial (34). Also, anti-inflammatory effects of a nitrate-rich diet have been reported for periodontal health (53). It has been reported that periodontitis recall patients presented a decrease in gingival inflammation after regular consumption of juice high in nitrate compared to a control group consuming juice without nitrate (54, 55).

Bacterial fermentation of proteins is complex (Figure 1). In the gingival area, there is absence of sugars but access to proteins/peptides from gingival crevicular fluid (GCF). In particular, under inflammatory conditions, the flow of GCF leaking out through the very thin (4–5 cells) epithelial barrier, is increased (56). The junctional pocket epithelium is non-keratinized, which allows migration of cells such as neutrophils and macrophages by chemotaxis. Thus, GCF, inflammatory cells and desquamated epithelial cells are the main sources of nutrients for the subgingival plaque bacteria. GCF is a serum exudate that contains components found in serum such as proteins, peptides, amino acids, hormones, and vitamins (57). The levels of sugars are negligible in the serum (except in glycemic diabetic patients) and the level of fermentation of carbohydrates/sugars in the subgingival environment is thus low. The fermentation of proteins is, therefore, the predominant metabolic pathway and this favors the proteolytic species in the oral microbiota (secondary fermenters, Figure 1). Nitrogenous compounds can be degraded into short chain fatty acids (SCFAs), NH_3_, sulfur compounds, and amines by the bacteria of the subgingival biofilm. It should also be noted that urea originates from serum *via* GCF (58). Therefore, the production of NH_3_ from urea is mainly occurring in the dental plaque, and the subgingival plaque in particular.

The nutrients present in the oral cavity are further used by secondary fermenters, predominately anaerobic and asaccharolytic bacteria, which collectively produce numerous metabolites, such as SCFAs, amines and gases (Table 1). While most of the metabolites, produced are further used by the microbial community, others are excreted as waste products. This cascade of metabolites increases when the activities and growth rates of the bacteria are increased. The gingival crevice is inaccessible and partly sequestered from the influence of saliva and the conditions prevailing above the gingival margin.

The homofermentative bacteria of the phylum Firmicutes (*Streptococcus, Gemella, Granulicatella*), are characterized as producing mainly lactic acid from carbohydrates and as being the most abundant bacteria in the human oral cavity. Other primary fermenters, such as lactobacilli, bifidobacteria and clostridia, are prominent fermenters in the proximal part of the intestine but normally constitute only a small fraction of the oral microbiota. Instead, other heterofermentative species with adhesion properties, such as *Actinomyces, Corynebacteria, Rothia, Haemophilus, Neisseria, Prevotella* and *Fusobacterium* spp., are better suited to the conditions found in the oral cavity.

Secondary fermenters are predominantly anaerobic bacteria (Figure 1) that require a low redox potential for growth, but these are sensitive to oxidizing substances (oxygen, peroxides, oxygen radicals, isothiocyanate, NO) and can survive and grow only in “hidden areas”, such as the dorsum of the tongue and the interproximal areas between teeth and in and around the gingival crevice. Although many of these anaerobic bacteria can degrade both carbohydrates and proteins, they primarily metabolize monosaccharides and peptides/amino acids and are, therefore, dependent upon the co-operation activities and food chains in a polymicrobial community. There are numerous examples from the oral microbiota of microbial growth only in co-culture with other microorganisms, as well as examples of bacteria providing growth factors to their neighbors (132). Among the secondary fermenters, asaccharolytic bacteria are also found. *Veillonella* spp. use lactate as a nutrient and have been attributed an important role in producing weaker acids and contributing to neutralizing the pH in dental biofilms (37).

In the subgingival microflora, several proteolytic species (*Porphyromonas* spp. and *Campylobacter* spp.), are asaccharolytic, using peptides/amino acids for their energy supply. Most of the amino acids that can be utilized are also essential for growth of the anaerobes. The fermentation process performed by the anaerobes is complex in that it involves extensive degradation of proteins, peptides, and amino acids. The subgingival microbiota constitutes an efficient fermenter (Figure 1), generating plentiful well-known and less-known bioactive metabolites with wide-ranging effects on the gingival tissues (18, 133, 134). These metabolites include an extensive collection of SCFAs, amines, and various gases (Table 1). The impact of these molecules are often crucial for the host, being beneficial at low and detrimental at high concentrations.

SCFAs, which include formic acid, acetic acid, propionic acid, butyric acid and valeric acid, are produced from carbohydrate fermentation. They are also produced during the proteolytic degradation carried out by anaerobic gut bacteria, as well as oral bacteria (62, 135). SCFAs play important roles in both the maintenance of health and the development of inflammatory diseases (18, 136). They exhibit antimicrobial activities against specific bacteria in the microbial community (69). SCFAs (primarily acetate), are extensively produced by *F. nucleatum* and *P. gingivalis* (63, 64, 66). Butyric acid is produced during the fermentation of glutamate and has an unpleasant odor (18). A mean concentration of 2.6 mM of butyric acid has been reported in subgingival plaque of periodontitis patients, and 9.5 mM propionic acid, while these SCFAs were below the detection limit at healthy sites (68). In GCF high mean values have been reported, 3.11 mM for butyric and 11.68 mM for propionic acid respectively (67). Furthermore, it has been shown that the concentrations of butyric acid, propionic acid, acetic acid, and isovaleric acid in GCF are significantly reduced two weeks after treatment of periodontitis affected sites (67, 137). Thus, it appears that the concentrations of SCFAs correlate with gingival inflammation (138).

The amines indole and skatole are converted from the amino acid tryptophan during fermentation by several anaerobic oral bacteria, e.g., *Fusobacterium*, *Prevotella*, and *Porphyromonas,* present within the subgingival microbiota (64, 74–76, 78). However, few studies have examined the presence and amounts of indole and skatole in the oral cavity. Other bioactive amines, the polyamines putrescine, spermine and spermidine, have not only been detected in GCF of periodontitis-affected sites but also shown to decrease after treatment (80, 139). Concentrations of 2.76 mM of putrescine have been measured (80). Cadaverine has been investigated in saliva and been shown to increase in the absence of oral hygiene measures (140).

**Figure 2:**
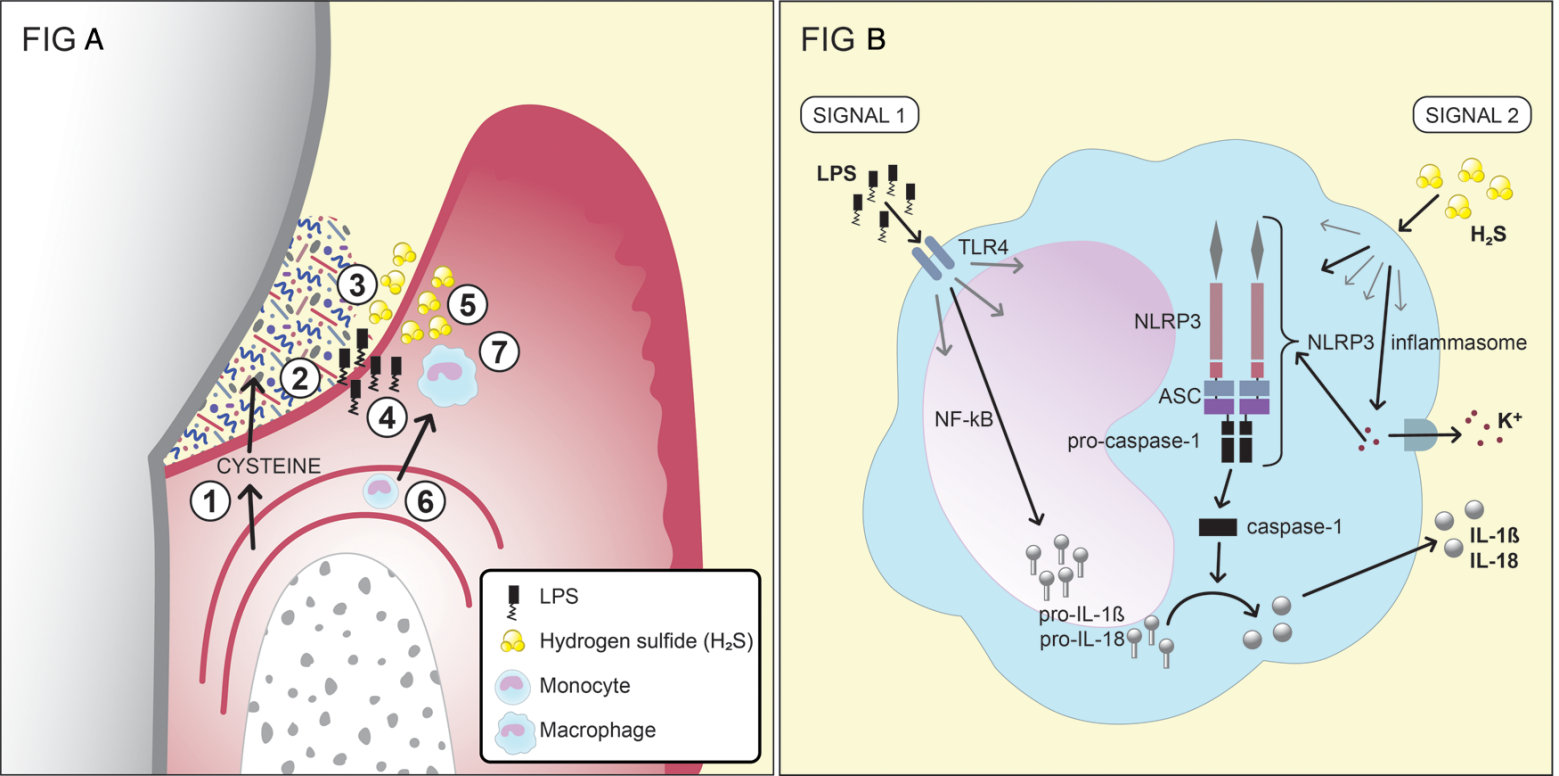
(**A**) A schematic of the production and effects of the bacterial metabolite H_2_S in the inflamed gingival pocket (reprinted from hydrogen sulfide exposure induces NLRP3 inflammasome-dependent IL-1β and IL-18 secretion in human mononuclear leukocytes in vitro by Basic A, Alizadehgharib S, Dahlén G, Dahlgren U in clin. Exp. Dent. Res. 2017;3:115–120. https://doi.org/10.1002/cre2.69). 1. Serum exudate from blood vessels containing serum proteins, peptides and amino acids, including cysteine. 2. The exudate (gingival crevicular fluid; GCF) passes through the thin pocket epithelium (junctional epithelium) into the subgingival pocket. 3. The subgingival plaque, which contains numerous, mainly Gram-negative, anaerobic bacteria with capacities to degrade proteins, peptides and amino acids, including cysteine. 4. Growing Gram-negative anaerobes release lipopolysaccharides (LPS), which cross the junctional epithelium into the gingival connective tissues. 5. Growing Gram-negative anaerobes (Fusobacterium spp., P. gingivalis, Treponema spp., and others) produce metabolites, e.g., hydrogen sulfide (H_2_S). 6. The inflammatory lesion attracts monocytes, which migrate into the connective tissue and differentiate into macrophages. 7. The effects of LPS and H_2_S on macrophages and the subsequent production of the pro-inflammatory cytokines IL-1β and IL-18. This figure has previously been published in an Open Access journal, distributed under the terms of the Creative Commons Attribution ver. 4.0 International License (https://creativecommons.org/licenses/by/4.0/), which permits use, distribution and reproduction in any medium, provided the original work is properly cited. (**B**) The two signals that lead to the production and secretion of the IL-1β and IL-18 by monocytes/macrophages. For the first signal, the production of pro-IL-1β and pro-IL-18 is stimulated by exposure of the monocytes/macrophages to LPS. The second signal, which can be induced by H_2_S, results in the release of the two cytokines from the cell after the formation of the NLRP3 inflammasome.

Gases, including carbon monoxide (CO), hydrogen gas (H_2_), NO, NH_3_, and hydrogen sulfide (H_2_S), are critical end-products of the fermentation processes. CO and carbon dioxide (CO_2_) are main sources for carbon, and even essential for some bacteria, e.g., *Capnocytophaga* spp (98). H_2_ is important for many microorganisms (e.g., *Campylobacter* spp. and *Archaea*) for their survival and growth. H_2_S is produced by bacterial fermentation of the sulfur-containing amino acid cysteine by mainly anaerobic species associated with periodontal diseases, including *Porphyromonas* spp., *Fusobacterium* spp., *Alloprevotella tannerae*, *Parvimonas micra*, *T. denticola*, *V. parvula*, and *S. anginosus* (101, 102, 141, 142). It can also be produced through the reduction of sulfate by the sulfate-reducing bacteria *Desulfovibrio* spp., *Desulfobacter* spp., and *Desulfomicrobium orale*, which may also be present in periodontal pockets (103, 104). Its smell resembles that of rotten eggs, and it is detectable from a concentration of 0.01 ppm in air. H_2_S, together with methyl mercaptan (CH_3_SH) and dimethyl disulfide (CH_3_SSCH_3_), are important contributors to oral malodor and are usually referred to as volatile sulfur compounds (143, 144). The highest concentrations of H_2_S reported from diseased periodontal pockets are in the range of 1.5–1.9 mM (109, 110). It has been shown that the subgingival levels of H_2_S are higher at diseased sites than at healthy sites, as are the capacities of subgingival microbial samples to produce H_2_S from cysteine (120, 145–147). Thus, the direct measurements of H_2_S and indirect measurements of the capacity of the microbiota to produce H_2_S are consistent with each other, in that the more dysbiotic the microflora, the higher is its production of H_2_S. In addition, accessibility to cysteine is expected to be greater at diseased sites due to the increased secretion of GCF, which contains cysteine. The concentration of cysteine in the serum of healthy blood donors has been reported as 0.26 mM, and it is likely that its level in the GCF is of a similar magnitude (148). Based on these observations, H_2_S has been suggested to participate in the pathogenesis of periodontal disease (Figure 2A) (110, 120, 121, 147, 149).

As for bacterial production of H_2_S, the enzymes involved also catalyze the production of other by-products, such as pyruvate, NH_3_, and serine (92, 95, 150). NH_3_ is produced by several bacteria associated with periodontitis, such as *Prevotella* spp., and *Fusobacterium* spp. and asserted to contribute to the slightly alkali subgingival environment (65). NH_3_ can, apart from proteolysis, also be produced from nitrate, as has been shown in saliva and tongue scrapings (139). There are however, to our knowledge no reports to date of measurements of NH_3_ in periodontal pockets.

Methanogenesis is the process in which methane is produced and usually the final step in the fermentation of organic materials. Methanogenesis takes place only under anaerobic conditions by *Archaea. Archaea* is regularly missed in culture studies but archaeal DNA can be detected by PCR amplification and by sequencing of subgingival plaque from most adults (151). Especially methane-producing *Archaea,* e.g., *Methanobrevibacter* spp., have gained interest in the ecology of the oral microbiota because of their requirement for H_2_ produced from fermentation by the oral microbiota, e.g., *Campylobacter* spp. (Figure 1) (152).

## 3. Host response

The host response to the microbial challenge in periodontal disease is complex and numerous factors are at play (23). It should be noted that the connection between the gingival epithelium and the tooth surface is vulnerable for an exposition of microorganisms of the dental biofilm and their metabolites. The epithelial barrier is extremely thin, non-keratinized and without a mucin layer and is easily penetrable for small molecules such as many of the bacterial metabolites. The host defense in the gingival pocket is supported by the gingival exudate containing serum defense factors such as immunoglobulins and complement factors as well as phagocyting cells such as neutrophils and macrophages. At prolonged exposition of the gingiva for the dental biofilm, an immune-response phase involving lymphocytes and plasma cells, is activated. In contrast to neutrophils and macrophages, which are rapidly and constantly recruited by chemotaxis through the pocket epithelium, lymphocytes and plasma cells are recruited at a later point into the connective tissue beneath the pocket epithelium (inflammatory connective tissue). When activated, these cells produce and release a cascade of cytokines characteristic for a more chronic type of inflammation occurring in long-standing gingivitis. The host response to the dental biofilm is balanced (homeostasis) against the bacterial challenge and the activity in the dental biofilm, characterized by the chronic inflammatory phase, and due to continuous fluctuation of the bacterial activity within the dental biofilm the response includes various degrees of acute phases. In addition, the host is under constant and challenging influence from external and internal host-related factors often referred to as host susceptibility for the tissue breakdown (153). The factors included are systemic diseases (e.g., diabetes), medications (cytostatic drugs), smoking and psychological factors (stress, allostatic load), which results in a highly unpredictable outcome of the inflammatory response at the individual level, after many years of bacterial challenge (23, 154–159).

## 4. Metabolites in the bacteria-host interplay

The many and diverse bacterial waste products in the periodontal pocket are assumed to be abundant due to the lack of salivary cleansing that is seen above the gingival margin. Thus, subgingivally the high concentrations of the waste products of bacterial proteolysis results in a vulnerable oral niche. Apart from potential toxic properties of the waste products at high concentrations, many of them are also present in the human host and may have various functions and contribute to diverse effects on the cells of the host. Several metabolites function as signaling molecules and can trigger or dampen the inflammatory response of the host. It should, however, be highlighted that details and mechanisms known to date are almost exclusively based on *in vitro* studies and thus, the importance and relevance of these metabolites *in vivo* are still mostly undetermined. Therefore, more studies are warranted to delineate their relevance in the pathogenesis of periodontal diseases.

### 4.1. Effects on the bacteria/biofilm

The production of the various metabolites is mainly the result of bacterial fermentation for energy recovery, although it also acts to scavenge materials for protein synthesis. This process probably affects the local environment in the biofilm and, thus, may in higher concentrations be harmful to bacterial cells (111, 129). It appears that bacteria can regulate this activity by adjusting the expression of enzymes. This has been shown for H_2_S where the presence of high concentrations of cysteine in the culture broth during the cultivation of *Fusobacterium* spp., resulted in downregulation of H_2_S-producing enzymes (105). Similarly, high concentrations of H_2_S have been shown to suppress the sulfate uptake and H_2_S production of sulfate-reducing *Desulfovibrio* spp (160).

Analyzing from a bacterial point of view, the various metabolites are not only waste products of fermentation but can have protective functions as well. Many of the metabolites, including NH_3_, spermine, spermidine, H_2_S, and NO, have been shown to increase bacterial resistance to antibiotics (81, 88, 112, 113, 130). In addition, increased resistance to leukocyte-mediated killing has been described for H_2_S-producing bacterial strains or strains grown in the presence of H_2_S, as compared to H_2_S-deficient strains or strains grown in the absence of H_2_S (114, 115). There is also evidence that H_2_S production is advantageous for the bacteria, in that bacterial H_2_S protects the bacteria from oxidative stress (116). Furthermore, impairment of neutrophil function has been reported for another bacterial metabolite, NH_3_ (89).

A number of various factors such as bacterial virulence factors (bacterial survival factors such as adhesins, capsule, leukotoxin) participate in the regulation of the biofilm community in addition to the various metabolites formed (161, 162). A beneficial outcome for the bacteria is the reported antimicrobial effect of various metabolites in the competition and cooperation with other species (CO, SCFAs, nitrite, NO) (33, 69, 73, 94, 131). Interestingly, it appears that bacterial producers of various fatty acids are not themselves affected by the acids produced, but the growth of other microorganisms, indicating a competitive advantage for SCFAs-producers in the biofilm (69). Moreover, a synergistic antimicrobial effect *in vitro* for combinations of fatty acids has been reported (73). Possibly, bacterial metabolites are also involved in the formations of, but also interspecies interactions in, dental biofilms (82, 117, 125).

### 4.2. Toxic consequences for the host

Plentiful of our knowledge on various gaseous metabolites is based on initial studies of human exposure to very high concentrations, shown to be hazardous to humans (163, 164). H_2_S, as an example, has previously been in focus as an industrial product in oil refining and mining, resulting in environments with high concentrations of H_2_S in the air. Also, CO_2_ is a common industrial gas used in many beverages, and fire extinguishers, among others (99). At high concentrations, both H_2_S and CO_2_ have toxic effects with life-threating consequences. A H_2_S concentration of 100 ppm in air irritates the eyes, and at concentrations of ≥300 ppm there is loss of consciousness, respiratory failure and, potentially, death (118, 119). Concentrations above 10% CO_2_ in air is comparably hazardous (99). Also, reported exposures to CO and NH_3_ have similar consequences where inhalation is a common exposure route primarily affecting the respiratory system of the host (90, 96, 97, 163, 165). Both the concentration and the duration of exposure are important factors to consider when evaluating the health effects of these metabolites. Furthermore, other metabolites have been reported toxic when ingested in high amounts, such as amines in fermented foods causing food poisoning, but also nitrate which is converted to nitrite and in high amounts may result in methemoglobinemia in children (85, 166). Whether the metabolites produced in periodontal pockets reach these high local concentrations and exposure times, with potential toxic consequences on host cells, is still undetermined. Theoretically, toxic amounts of the metabolites may potentially result in necrosis of epithelial cells, resulting in bacterial invasions and subsequent infections, clinically manifested as abscesses. Nevertheless, little is known about the concentrations and variations within the periodontal pockets, mainly due to the lack of flexible and reliable methods for estimation of the subgingival metabolites.

### 4.3. From metabolites to signaling molecules

The gaseous metabolites, CO, NO, and H_2_S are all produced by the endogenous cells of humans and animals, and function as signaling molecules, with biological effects evident at low doses (96, 167). In the catabolism of heme, CO is produced, approximately 18 μmol/hour (167). In serum, a concentration of 24 μmol/L has been reported for NO in healthy adults (168). Correspondingly, micromolar concentrations of H_2_S have been detected in the blood and in brain tissue (119, 169, 170). Among many diverse functions, it has been shown that exposure to CO has neuroprotective effects, that NO regulates blood pressure, and that H_2_S can vasodilate blood vessels and act as a neurotransmitter in the brain (96, 171–173). The signaling molecules have been implicated in stroke, Dowńs syndrome and Alzheimeŕs disease, among other disorders of the CNS (96, 171). Hence, the hormesis phenomenon, where low doses of a toxic agent are beneficial, can probably be applied on many of the metabolites of the fermentation occurring in periodontal pockets.

So, not only the colonizing bacteria but also the host cells possess CO-producing/NO-producing/H_2_S -producing enzymes. NO is produced by NO synthase enzymes (NOS) (174). In humans and animals, three H_2_S-producing enzymes have been identified: cystathionine-β-synthase, cystathionine-γ-lyase, and 3-mercapto-sulfurtransferase (175). These enzymes are present in the majority of human cells, including those in the periodontium (122, 176). Similarly, amines are produced by colonizing bacteria and host cells, but may also be derived from foods (86).

The exact mechanisms by which the bacterial metabolites affect human and bacterial cells are still not fully understood for the majority of them. It is for instance regarded that NH_3_ can easily penetrate cell membranes and alter intracellular pH (177). It has been reported that the ability of H_2_S to split disulfide bonds is an important factor for its activity. This cleavage may, by reducing binding to cell surface antigens, contribute to the inhibition of antibody-mediated immune responses reported in the presence of H_2_S (115). In addition, H_2_S is a known inhibitor of cytochrome c oxidase (178, 179). Three biochemical pathways for H_2_S signaling have been described. H_2_S can react with metal centers, modulate the cysteine residues of proteins by S-sulfhydration, and can react with reactive oxygen and nitrogen species (150, 180, 181). The fact that H_2_S binds to metals has been exploited for the detection of H_2_S production with lead-acetate impregnated strips (182). H_2_S also functions as a reducing agent (92, 183). Furthermore, it has been reported that H_2_S can pass through cell membranes due to its solubility in lipids (92, 119, 170). HS^−^ ions use the AE-1 channel to pass through the cell membrane in mammals, whereas a hydrosulfide ion channel has been described for bacteria (146, 184).

Of the SCFAs, butyric acid is extensively studied, not only in the oral cavity but also in the intestine (71). It is considered a biologically active signaling component with both anti-inflammatory and inflammation-promoting effects on host cells (70, 71). Butyrate has been shown to have different effects on different cell types, in what is known as the “butyrate paradox”, whereby the presence of butyrate promotes healthy cells in the colon and simultaneously inhibits cancerous cells (185). This phenomenon is still not fully understood. Butyric acid has been reported to be present at increased levels at diseased periodontal sites, and *in vitro* studies have shown that butyric acid can induce the apoptosis of monocytes and fibroblasts, among other host cells (62, 68, 186–189).

### 4.4. Anti—and/or pro-inflammatory consequences

That numerous metabolites are also signaling substances implies that they affect host cells in various ways. These effects are not just connected to the endogenously produced amounts, but also to the quantity of the metabolites produced by the bacteria colonizing the different body compartments. It has been shown that several metabolites, in diverse both pro- and anti-inflammatory manners, affect several host cell types (70, 71, 100, 122, 164, 190, 191). These immunomodulatory consequences have been comprehensively reviewed for H_2_S elsewhere (122). Briefly, the anti-inflammatory effects of H_2_S on neutrophils include inhibition of neutrophil adhesion, neutrophil extracellular trap (NET) formation, and myeloperoxidase secretion, as well as the release of other contents of the neutrophil granules (122, 192, 193). Furthermore, monocytes/macrophages have been reported to be affected by H_2_S exposure in an anti-inflammatory manner by promoting macrophage polarization from the M1 type to the more anti-inflammatory M2 type (194). In addition, H_2_S can inhibit the secretion of pro-inflammatory cytokines and stimulate the secretion of the anti-inflammatory IL-10 (195). This inhibition and secretion has also been shown for CO (96).

In contrast, exposure to H_2_S *in vitro* has been shown to induce: apoptosis in human periodontal ligament cells and human gingival fibroblasts; the secretion of IL-6 and IL-8 from these cells; and IL-8 secretion from epithelial cells (196–198). Moreover, cell death and functional inhibition have been reported for lymphocytes exposed to H_2_S (199). The *in vitro* exposure of a monocyte cell line to H_2_S has been shown to result in the secretion of the pro-inflammatory cytokines IL-1β and IL-18, in a process that requires two signals. The first signal generates the production of pro-IL-1β and pro-IL-18 in the nucleus, while the second signal activates the NLRP3 inflammasome and induces the secretion of the cytokines from the cell (Figure 2B). The NLRP3 inflammasome is a protein complex that, when formed, cleaves the inactive forms of the proteins, i.e., pro-IL-1β and pro-IL-18, to IL-1β and IL-18 (200). The first signal may be activated by LPS, although there is evidence that H_2_S can also contribute to this first signal (121). The second signal, leading to the formation of the NLRP3 inflammasome, can be induced by various substances, such as peptidoglycans and ATP, but also by H_2_S (120, 121). The results from a clinical study involving periodontitis patients and healthy controls showed differences between the two groups in terms of the patterns of cytokine expression from monocytes exposed to H_2_S (120). Various immunomodulatory roles of H_2_S in the human body are not exclusive for H_2_S but also seen for other metabolites. SCFAs, for instance, have been shown capable of recruiting neutrophils to the GCF though binding to the neutrophil-specific chemotactic receptor FFA2R (63, 66). NH_3_ has been reported to affect the functions of neutrophils, such as their affinity of receptors, their phagocytosis, degranulation, and metabolism (89, 201).

Given that bacterial production of H_2_S in the periodontal pocket promotes the inflammatory response of the host to the microbiota, it can be regarded as both detrimental (increased secretion of pro-inflammatory cytokines) and beneficial (increased GCF and, consequently, higher levels of nutrients in the periodontal pocket) for the bacteria. Additional activities of H_2_S, e.g., in enhancing bacterial resistance to antibiotics and attenuating leukocyte-mediated killing of bacteria, are beneficial for the bacteria in the host-bacteria interplay (112, 114). In contrast, anti-inflammatory effects of endogenously produced H_2_S have been reported as favorable for the host (122). Since H_2_S is recognized by the host as an endogenous molecule, the inflammatory response may be exacerbated, resulting in chronic inflammation (as in periodontitis). Thus, the ultimate impact of H_2_S in the pathogenesis of periodontal diseases is difficult to determine due to the diverse effects of H_2_S on the bacteria and host cells. Furthermore, H_2_S may be oxidized to polysulfides or other sulfur-containing compounds, which also affect host cells, upon entering the host (95, 202, 203). A further complication is that other sulfur-containing metabolites, such as methyl mercaptan from methionine, are produced simultaneously. Moreover, the continuously fluctuating growth rates and activities of the microbes result in variable production levels of the various metabolites in the periodontal pocket. Collectively, these issues illustrate the difficulties associated with studying H_2_S, but also other metabolites, in the host-bacteria interplay and its roles in periodontal diseases.

## 5. The subgingival biofilm in destructive periodontal disease

Microbial metabolites are the main contributors to evoke an inflammatory response in the gingival tissues. The metabolic pathways in the biofilm are generally shared between different microbial species representing diverse genera. The metabolism of the biofilm is characterized by fluctuations between burst of activities and resting (dormant) microbial cells. The release of metabolites is constantly fluctuating, which normally and in a seemingly homeostatic condition is balanced by the host response inducing and maintaining an inflammatory process seen clinically as gingivitis. This inflammatory response should be regarded as a natural, protective response to a subgingival microbiota that is adapted and thrive in the inflamed and deepened gingival pocket which offers a perfect ecological niche (161). Simply, this is a normal condition occurring in everybody in absence of careful oral hygiene measures. Like the dripping water that cavitates the stone, loss of clinical attachment at long-standing inflammation is a slow process that becomes clinically visible after years of exposition as the host response and colonizing microorganisms increase in complexity as the disease progresses. The transition from gingivitis to destructive periodontitis has been a matter of various hypotheses over the years, involving both biofilm and host factors, and there are advocates for it primarily being a natural aging phenomenon (15, 23, 204, 205). The likelihood of progression or regression can be influenced by various determinants, (e.g., specific bacteria), but these processes will nevertheless occur in the absence of such factors (17). From this it follows that destructive periodontitis can occur in everybody with time, but the probability of disease to occur can be reduced and controlled by measures that reduce the risk, such as for instance daily tooth cleaning procedures that regularly disrupt the dental biofilm formed and decrease the bacterial load and the levels of released metabolic waste products. It is the way the host and the tissues respond, which could be delayed or disproportionate of various reasons, that is responsible in the long-term for the disease progression, based on intrinsic (genetics, age) or acquired factors (smoking, systemic diseases) making the host and tissues susceptible to a variable degree (153, 159, 205, 206). The central role of inflammation in the pathogenesis of destructive periodontitis is generally accepted (204).

In addition to the metabolites, other factors of the bacteria connected to their characteristics, such as endotoxins, capsule formation, fimbriae but also their production of leukotoxin, outer membrane vesicles, bacteriocins and other antimicrobial peptides, are also to be considered in these interactions with the host, as we have reviewed elsewhere (23, 161). These “virulence” factors are of importance for the survival and growth of the bacteria, but their importance in the pathogenesis is undetermined (161).

## 6. Can microbial metabolites reveal periodontal disease activity?

The net outcome of the very complex interactions between numerous microorganisms in the dental biofilm, where it is impossible to perceive who is doing what, may be estimated by longitudinal and continuous measurement of these waste products, possibly by the use of cheap and simple chair-side methods. But if this is clinically relevant is still mostly unexplored territory.

The role of specific bacterial species may intermittently play a decisive part. So far, predictive studies, using specific risk marker bacteria, have only reached weak sensitivity and/or specificity to prospectively predict future progression on site/individual level, and these markers have not become widely used in current risk evaluation of periodontal diseases (207). However, the Jp2 clone of *A. actinomycetemcomitans* in populations of African origin is an exception from this by the finding that those individuals with this particular bacterial genotype have a 14 times higher risk for progression of periodontitis within 2 years (208, 209). The reason for the limits to use bacteria as risk markers is probably due to that the prevalence and abundance of bacteria are inadequate indirect measurements for their activity at a given time point (e.g., time of sampling) (138). To evaluate the metabolic activity by measuring the level of metabolites and the capacity of biofilms to produce these in the subgingival niche would thus be a more appropriate approach for identifying risk determinants, such as various metabolites, for the probability of disease progression, but this research area is still in its infancy. GCF has been used as a sampling source for biomarkers of periodontitis using bacterial metabolomics for diagnostics of the microbial and host interaction (210). In a systematic review on four metabolomic studies on biomarkers of GCF, it was shown among 40 significantly discriminant metabolites, predominantly related to amino acid and lipid degradation, that the diagnostic accuracy was low (211). New and more sophisticated molecular methods, such as the use of proteomics and metabolomics, will add knowledge in this area but due to often small sample sizes, also approaches fitting more prospective large-scale studies are needed. Here, initial controlled *in vitro* investigations of host cells to prolonged exposure to clinically relevant concentrations of various metabolites in an appropriate environment regarding temperature, pH, and oxygen level, may be followed by more advanced clinical studies using sophisticated molecular approaches.

Thus, studies on the major metabolites produced under bacterial activities (burst of activities) are suggested for future research on metabolic biomarkers. Simple and easily performed methods for the measurement of the metabolic load and the capacity to produce various metabolites (at rest and at challenge) experimentally *in vivo* or *ex vivo* are needed. Longitudinal follow-up studies using chair-side evaluations at many sampling sites, mapping the metabolic landscape in each individual, would disclose metabolic determinants in identifying individuals with a greater risk for disease progression.

## 7. Conclusion

The metabolites are the net outcome of the very complex interplay between numerous and different microorganisms in the dental biofilm. Much research has focused on the composition of the biofilms and the presence of certain periodontopathogens. This review focuses on the functions and dynamics of the biofilm, the metabolites, and their interplay with the host to better understand the impact of the biofilm in destructive periodontal disease. Here, simple chairside methods can complement molecular approaches and be applied longitudinally in clinical studies for determination of major metabolites and their clinical relevance on subjects with various periodontal conditions.
